# Where the Silky Cotton Grows

**Published:** 1894-12-29

**Authors:** 


### Studies in Applied Sociology.

"WHERE THE SILKY COTTON GROWS."

A generation ago there was war in the United States, and as a direct consequence there was starvation in Lancashire, for the close blockade kept by the North on the southern ports prevented any exportation of cotton save such as was brought in comparatively scanty quantities, and at risks which claimed their equivalent in price by venturesome privateers. What would be the result to-day if for any reason the American cotton crop were again to fail?

Bad enough, certainly, but no foreign country would suffer as England did during the "cotton famine." The southern states of North America are still the largest exporters of cotton, but no longer the only ones. Britain remains the largest consumer, and Britain imports less than it used to do. In 1892 England imported 11 per cent. less raw cotton than in the previous year, and paid 18 per cent. less for it. Probably the cheaper price compensated for the smaller quantity, and so we did not hear of "distress in the cotton districts." But the fact indicates that we have ceased to have the old monopoly of manufactured cotton. The United States are rightly encouraging the domestic industry, so are various European countries, but our enemy, if we dare call her so, is of our own household. India is becoming not merely a cotton-growing, but a cotton-manufacturing country. Not on all points, however, is there competition. The raw cotton of India is inferior to the best American, the fibre being shorter and coarser, therefore the finest goods will probably continue to be imported.

Less as a manufacturer than as an exporter of cotton is India to be feared; that is, we have less cause to dread her rivalry than has America. For Indian cotton is cheap, and for the coarser makes of stuff it is forcing its way into the market, not only in England, but in every cotton manufacturing country on the Continent. But another formidable rival has arisen of late years in Egypt, which twenty years ago sent out almost no cotton, and is now increasing her exports year by year. The quality of Egyptian cotton equals that of all but the very best American, and the best testimonial to its worth is that America itself is importing it in increasing quantities. In 1885—the first year of which we have a record—the United States imported 101 bales of Egyptian cotton, each bale weighing 710 pounds net. In 1893 they took nearly 40,000 bales. An American expert thus describes the Egyptian cotton: "Egyptian cotton has a long, strong, silky staple, from 1 to 1½ inches in length. It is specially adapted for thread, fine yarn, fine underwear, and hosiery, and for goods requiring smooth finish and high lustre. It gives to fabrics a soft finish, somewhat like silk goods, and this, together with its lustre, makes it desirable for use in cotton mixed silk goods. It is claimed that dyed and printed silk goods made of Egyptian cotton retain their colour and lustre longer than fabrics made of American upland cotton. The Egyptian cotton is not of as fine a quality as our South Carolina sea-island cotton, and, of course, does not command so high a price; but for such purposes as I have indicated it is better than American upland cotton, and it sells in Liverpool about one penny a pound higher." With Egypt and India, then, to supply us, and with such smaller quantities as we receive from Brazil and Peru, we need not fear a "cotton famine," even though, as is sometimes threatened, a "tariff war" should break out between us and America. And yet, in spite of this, it may be doubted if our cotton industry is not within measurable distance of decay. While we had the monopoly of good machinery and the best industrial methods we could carry things with a high hand, but these days are gone. The American cotton industry is as good, though not as large as our own. Some of our "cotton lords" have even found it to their advantage to set up mills in Rhode Island and New Jersey, in order to obtain a share of the American home trade. The Indian manufacture confines itself chiefly to spinning as yet, but it is not to be expected that it will permanently confine itself to doing only half the work necessary to convert the raw material into the finished product, ready for human needs. The words of a French Commission (Commission Permanente des Valeurs de Douane, 1893) are worth remembering in this connection: "The United States and British India are in an exceptionally favourable position in the cotton industry; both these countries are producers of cotton. They have the raw material at hand; both have an immense population, and consequently an illimitable field of consumption; the one has an inexhaustible supply of labour, the other has a genius for mechanical art; both have at their disposal immense capital. England and Continental Europe, in a period not far distant, must renounce a competition with the industry of these two countries in the markets which are found in their reciprocal spheres of action."

The great North American continent can for many years to come consume all the cotton it produces. Therefore the United States need grieve the less over the loss of another customer. Perhaps it matters more to us that Russia has joined the cotton-producing countries. The development is confined to Asiatic Russia, and is largely the result of artificial fostering. Nevertheless, it is so successful that restriction rather than encouragement will soon be needed. Already, according to the *British Trade Journal* of last November, "The Ferghana authorities require the cultivators to reserve one-fourth of their fields for the cultivation of cereals." Wheat, barley, and rice have increased in price in the province owing to the diminution in their production. European Russia imported from her Asiatic provinces and from Persia in 1889 no less than 38,000 tons, and the produce goes on increasing. Especially is this the case where American seed has been introduced, as this gives a finer product than the indigenous cotton. In 1889 the Tashkend crop thus raised amounted to 4,000 tons, in 1891 it had reached 16,340 tons—multiplied fourfold in two years. With this competition to face, it is not to be wondered at that the importof American cotton decreased, and the diminution—78,000,000 pounds—is almost identical with the quantity received from Asia. No doubt the preference for the native product is partly due to duties on imported goods, but this is a point which every exporting country must reckon with, and of which America has no right to complain. But it may be that the development of Russian cotton-growing is a less serious matter for South Carolina than it is for Lancashire.

